# Laparoscopic Transcystic SpyGlass Discover-Assisted Common Bile Duct Exploration and Clearance: An Efficient and Cost-Effective Alternative for Common Bile Duct Stone Management

**DOI:** 10.7759/cureus.80398

**Published:** 2025-03-11

**Authors:** Ahmed Abdelkader, Sanjay Basu, Atta Ul Aleem Khalid, Jameels Siddique, Aashir Luqhman, Waleed Naveed, Anupam Chandran, Georgy Thomas, Tasveer Javed, Antonio Golpe

**Affiliations:** 1 General Surgery, Scunthorpe General Hospital, Scunthorpe, GBR; 2 Upper Gastrointestinal Surgery, Scunthorpe General Hospital, Scunthorpe, GBR

**Keywords:** cbd stones, common bile duct (cbd), gallstone disease (gsd), laparoscopic cbd exploration, laparoscopic cholecystectomy, spyglass

## Abstract

Aim

This study aimed to assess the safety and efficacy of laparoscopic transcystic SpyGlass Discover (Boston Scientific Corporation, Marlborough, MA, USA) common bile duct (CBD) exploration combined with laparoscopic cholecystectomy (LC) as a single-stage procedure for patients requiring cholecystectomy and confirmed, suspected, or complex CBD stones. The study also evaluated the cost-effectiveness of this single-stage procedure compared to the two-stage endoscopic retrograde cholangiopancreatography (ERCP) followed by LC. The study adhered to the local clinical governance unit protocol and the Strengthening the Reporting of Cohort Studies in Surgery (STROCSS) guidelines.

Materials and methods

This is a retrospective cohort study conducted from January 2023 to July 2024. The safety, efficacy, and cost-effectiveness of SpyGlass Discover CBD exploration performed by a single consultant were evaluated. Of the sample population, 38 patients who underwent CBD stone management were included. Twenty-one patients (Group 1) had SpyGlass Discover CBD exploration planned, while 17 patients (Group 2) underwent the conventional preoperative ERCP followed by LC. All adult patients undergoing planned management were included. Data for comparison included age, gender, length of hospital stay, operative time, and success rates.

Results

Twenty-one patients underwent a single-stage operation using SpyGlass Discover. In nine cases, CBD exploration was performed, but no intervention was needed. In five cases, intervention was required, consisting of two laser lithotripsies and two mechanical lithotripsies with a basket. Seven cases had OTC, which showed a clear duct. The total operative time for Group 1 was 146 minutes on average. For those who did not require intervention, the mean operative time was 117 minutes, while the mean operative time for cases requiring intervention was 175 minutes. In Group 2, the mean operative time for ERCP + LC was 131 minutes. The length of hospital stay for Group 1 averaged 1.25 days, while Group 2 had an average of 1.52 days.

Conclusions

It was concluded that one-stage LC combined with SpyGlass Discover-assisted transcystic CBD clearance is an effective, safe, and cost-effective procedure when performed by skilled hands.

## Introduction

Gallstones are prevalent in the general population [1]. While 10% to 25% of people with gallstones may experience symptoms such as biliary colic and acute cholecystitis, and 1% to 2% may face serious complications [2-3], the majority of individuals with gallstones remain asymptomatic. These symptoms and complications often arise when stones enter the common bile duct (CBD), obstructing the bile flow to the small intestine. This can result in pain, jaundice, and, in some cases, cholangitis [4-5].

Laparoscopic cholecystectomy (LC) has been the gold standard for treating cholelithiasis since the early 1990s [6-7]. Additionally, isolated CBD stones are typically treated with endoscopic retrograde cholangiopancreatography (ERCP) [5]. However, opinions on the best approach to treating choledocholithiasis remain divided [8-9].

Preoperative ERCP followed by LC usually requires two rounds of anesthesia and two separate hospital stays. During the interval between operations, some patients may forgo LC, feeling satisfied with the results of the preoperative ERCP [10-11]. However, for both suspected and confirmed cases of CBD stones, one-stage procedures have shown distinct benefits. By addressing both conditions in a single operation with one anesthetic session, these methods are considered effective, safe, convenient, and well-accepted by patients [12-13].

Recent advancements have made laparoscopic transcystic CBD exploration and CBD stone removal possible through the use of the SpyGlass Discover cholangioscope (Boston Scientific Corporation, Marlborough, MA, USA) [14-18], which has an outer diameter of 3.5 mm. When used during elective LC (ELC) with intraoperative cholangiography, SpyGlass Discover allows concurrently treating CBD stones, shortening the time between diagnosing acute cholecystitis and performing ELC and improving patient outcomes. Additionally, altered gastrointestinal anatomy does not interfere with this approach [19]. The version used in this study, SpyGlass Discover, is designed for laparoscopic and percutaneous CBD interventions. The main difference from the SpyGlass DS, typically used for ERCP, is the catheter length: 65 cm for the Discover version.

This study aimed to assess the safety, efficacy, and cost-effectiveness of laparoscopic transcystic SpyGlass Discover-assisted CBD exploration when performed simultaneously with LC. The outcomes of this one-stage procedure were compared to the traditional two-stage method of ERCP followed by LC.

This article was previously presented as a meeting abstract at the 2025 ASIT Annual Scientific Conference on March 9, 2025.

## Materials and methods

Participants, interventions, comparisons, and outcomes (PICO) research question

Could laparoscopic transcystic CBD exploration with the assistance of SpyGlass Discover be an effective alternative to conventional techniques?

Study design and patient selection

We conducted a retrospective cross-sectional study following a predefined protocol recommended by the local Clinical Governance Unit. The study was reported in compliance with the Strengthening the Reporting of Cohort Studies in Surgery (STROCSS) guidelines for observational studies. Research ethics committee approval and patient consent were not required, as the study was retrospective and used unidentifiable data. The study was conducted in the general surgery department of Scunthorpe General Hospital, United Kingdom. We studied a sample population of 38 patients treated for CBD stone disease at our DGH between January 2023 and July 2024. The study focused on the efficacy of SpyGlass Discover CBD exploration and its cost-effectiveness. All patients had preoperative magnetic resonance cholangiopancreatography to identify CBD stones. The inclusion criteria for our study included all adult patients of any age or gender undergoing elective management for CBD and gallbladder stones. We excluded patients who received unplanned or emergency treatment.

Data collection

To ensure comprehensive data collection, a proforma was created for electronic use. This proforma was designed to collect the following data: age, gender, indications for SpyGlass Discover, interventions performed, length of hospital stay, total operative time, success rate, and complications. All patients underwent on-table cholangiography (OTC) before and after cannulation with SpyGlass Discover to identify filling defects. The procedure's success was defined by a clear CBD, as demonstrated on the postoperative OTC. All patients were monitored during the postoperative period and had a follow-up clinic appointment four to six weeks postoperatively to assess for complications. Cost-effectiveness was defined by the duration of hospital stay, total operative time, success rates, and the period of lost productivity.

Comparisons and outcomes

We aimed to synthesize outcomes for both groups, including comparisons of patient characteristics. The main outcome of this study was to assess the effectiveness, success rates, and cost implications of laparoscopic transcystic CBD exploration using SpyGlass Discover. These findings could have broader implications for the National Health Service (NHS) as a whole.

Data synthesis and statistical analyses

Categorical variables were summarized using absolute and relative frequencies and compared using the Chi-square test. Continuous variables were summarized as mean ± standard deviation and median (interquartile range), with the Kruskal-Wallis test comparisons. All statistical tests were two-tailed, and statistical significance was p<0.05. Statistical analyses were conducted using SPSS Statistics version 25 (IBM Corp. Released 2017. IBM SPSS Statistics for Windows, Version 25.0. Armonk, NY: IBM Corp.).

## Results

A total of 38 patients were managed for CBD stone disease between January 2023 and July 2024. Of these, 21 patients (39%) had planned laparoscopic transcystic CBD exploration using SpyGlass Discover, while 17 patients (61%) underwent the conventional preoperative ERCP followed by LC. The median age for Group 1 was 41 years, and Group 2's was 47 years. In Group 1, 39% of patients were male, compared to 61% in Group 2 (Table 1). The reasons for using laparoscopic transcystic CBD exploration with SpyGlass Discover included unsuccessful ERCP, contraindications to ERCP, or patient preference. The size and number of CBD stones were not factors in deciding between the two procedures.

**Table 1:** Demographic data *KW test (H)*: The KW test (H) was performed to compare the median age across the two groups. The p-value (0.92095) indicates no significant difference in age between Group 1 and Group 2. *Chi-square (χ²)*: A Chi-square test examined the association between sex and group allocation. The p-value (0.292953) suggests no significant difference in sex distribution between the groups. SD: standard deviation, IQR: interquartile range, KW test: Kruskal-Wallis test

Parameters	Total	Group 1	Group 2
Number of cases	38	21 (55%)	17 (45%)
Age			
Mean	50.54	49.78	50.31
SD	16.41	18.44	15.14
Median	47	41	47
IQR	32	32	25
KW test (H)	0.0098	p=0.92095	-
Sex			
Male	15 (39%)	7	8
Female	23 (61%)	14	9
Chi-square (χ²)	1.106	p=0.292953	-

**Figure 1:**
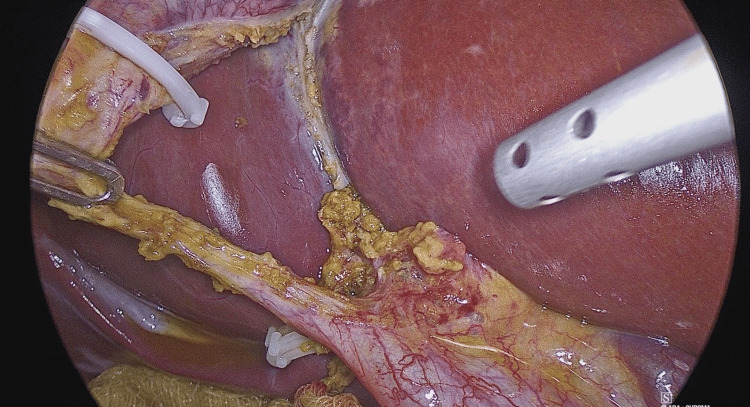
Extended critical view of safety

The standard LC procedure is typically initiated by creating a large posterior window (extended critical view of safety) to clearly identify the cystic duct and artery while ensuring that liver structures are excluded (Figure 1). Once Calot's triangle is identified, OTC is performed to clarify the biliary anatomy, identify any filling defects, and avoid unnecessary intervention in the biliary tree. This involves making a horizontal incision on the cystic duct to introduce the OTC catheter.

**Figure 2:**
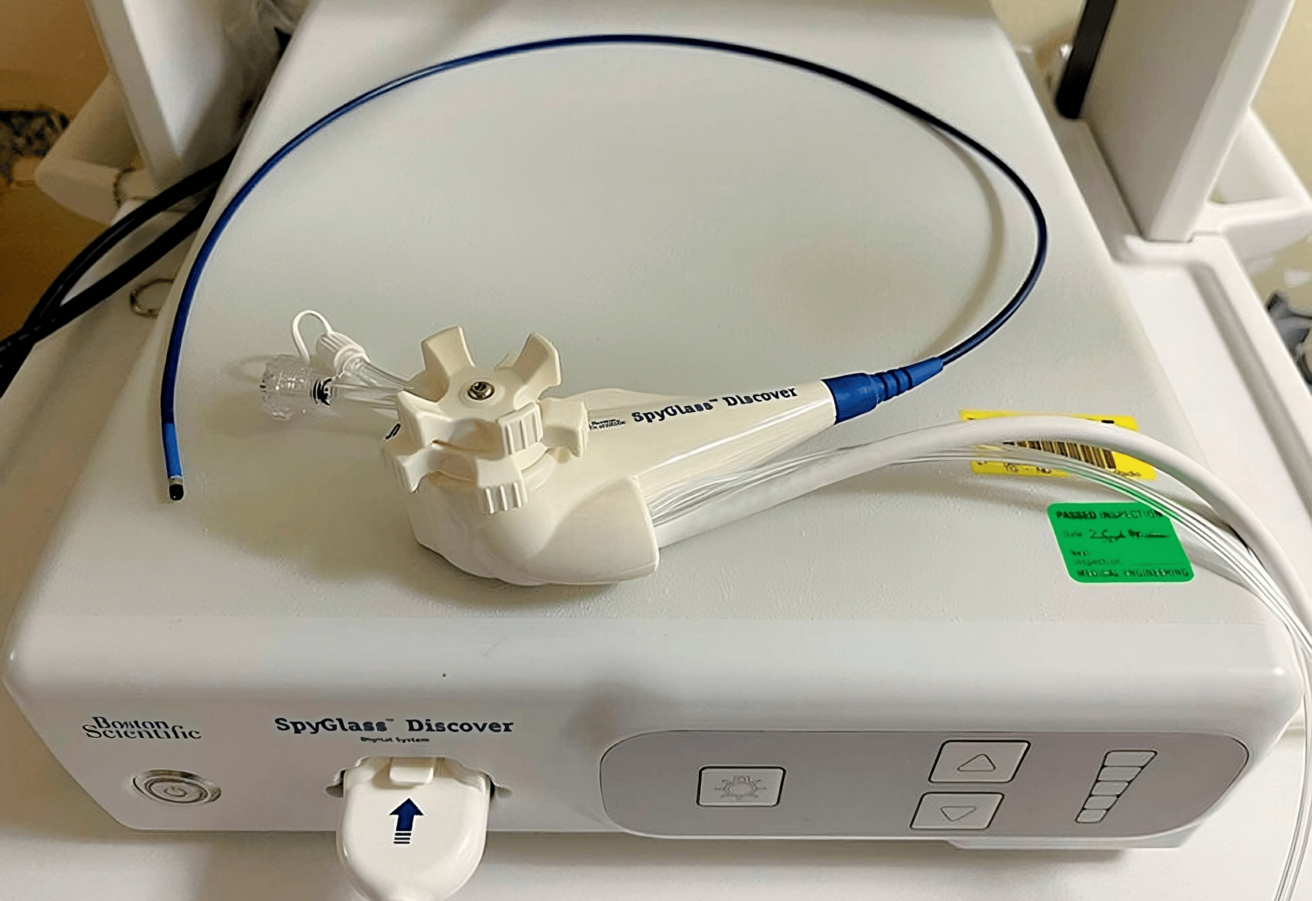
SpyGlass Discover

**Figure 3:**
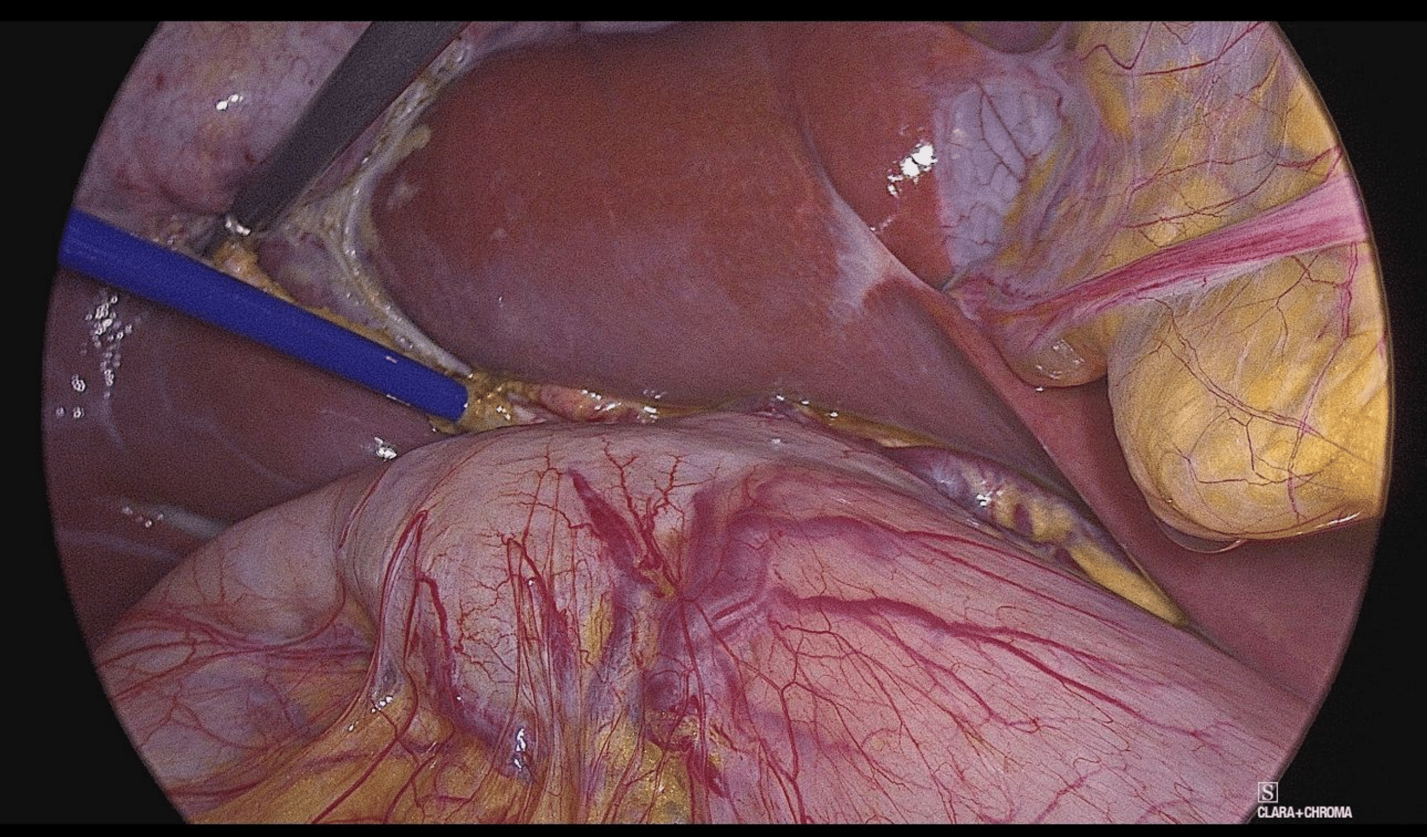
SpyGlass Discover catheter transcystic CBD cannulation CBD: common bile duct

In a smaller subset of cases (seven cases, 33%), OTC and flushing of the CBD were sufficient to remove microlithiasis. However, in most cases (14 cases, 67%), the SpyGlass Discover system was utilized (Figures 2-3). The SpyGlass Discover catheter, which is 65 cm long, allows for direct visual exploration of the CBD. Among these cases, nine (43%) involved small stones that were pushed into the duodenum using pressure washing, while the remaining five cases (24%) required basket removal (Video 1), mechanical lithotripsy, or laser fragmentation for larger stones (Video 2).

**Video 1:** SpyGlass Discover-assisted basket removal of CBD stones CBD: common bile duct

**Video 2:** Laser fragmentation

After stone removal, transcystic OTC was performed to confirm that the CBD was fully cleared and that there was no bile leakage. Finally, the cystic duct was sealed with a clip, and the cholecystectomy was completed. All videos and figures were taken from our original work in the general surgery department.

Access to the SpyGlass Discover system in cases with small CBD stones encouraged a surgical approach and helped avoid unnecessary ERCPs. This is because, after OTC, if any CBD stones were found that were difficult to flush with the OTC catheter, the SpyGlass Discover system provided multiple options for effective management.

**Figure 4:**
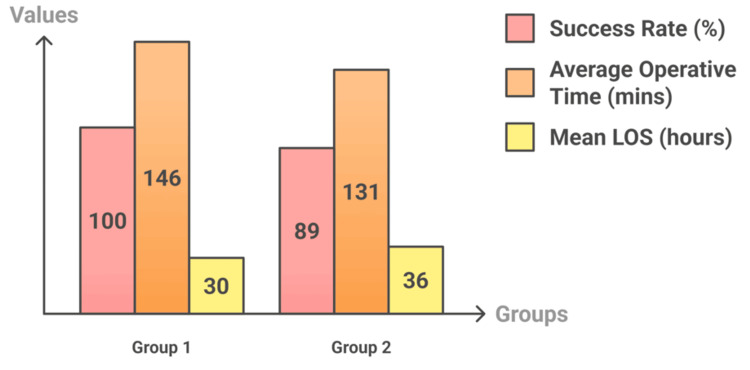
Comparison of outcomes between Group 1 and Group 2 LOS: length of stay

The outcomes showed that Group 1 had a mean length of stay (LOS) of 1.25 days, while Group 2 had a mean LOS of 1.52 days, including the ERCP and LC admissions. The average total operative time for Group 1 was 146 minutes, broken down into 117 minutes for cases that didn’t require interventions and 175 minutes for those that did. In comparison, the operative time for Group 2 (including both ERCP and LC) was 131 minutes (Figure 4).

The success rate for Group 1 was 100%, while Group 2 had a success rate of 89%, with two patients requiring additional ERCP sessions. No complications were recorded (Table 2).

**Table 2:** Comparison of outcomes OT: operative time, LOS: length of stay

Parameters	Group 1	Group 2
Mean OT (min)	146	131
mean LOS (days)	1.25	1.52
Success rate	100%	89%
complications	None	None

Cost comparison

According to the NHS payment scheme for 2024/25, an LC costs £2,500, while the average cost of an ERCP is £1,300. Laparoscopic transcystic CBD exploration using the SpyGlass Discover system was introduced as a new surgical procedure in our department, with an initial cost of £1,500 per case, which would be reduced to £1,000 if more than 10 cases were performed.

The cost comparison favored laparoscopic transcystic CBD exploration with SpyGlass Discover, despite its longer average operative time, due to shorter hospital stays, lower procedure costs, and higher success rates. Furthermore, the cost of lost productivity for Group 2 should be considered, considering the time between ERCP and LC. The operative time is expected to decrease as the procedure is performed more frequently in the department.

## Discussion

There is a growing trend toward CBD exploration as surgeons increasingly opt for the direct management of choledocholithiasis by clearing the CBD [20].

In a prospective, two-year study of 2,347 patients from 17 institutions, 9.8% experienced post-ERCP complications, with pancreatitis (5.4%) and hemorrhage (2%) being the most common. A Chinese study of 3,178 patients who underwent ERCP reported an overall complication rate of 7.9%, while a British study of 4,561 patients found a complication rate of 5% [5].

Retrospective studies show similar post-ERCP complication rates. A study of 16,855 patients undergoing ERCP from 1977 to 2006 found a post-ERCP complication rate of 6.85%, with the majority (5.17%) of complications being mild. High-risk patients and those undergoing more complex interventions have been found to experience higher complication rates [21-23].

In a prospective study of 707 patients undergoing CBD stone management with preoperative ERCP, 22% of the ERCPs were negative for CBD stones or sludge, despite following ASGE risk stratification criteria and pre-procedural imaging. Additionally, 12% of patients developed ERCP-related complications, including pancreatitis, bleeding, perforation, cholangitis, and anesthesia-related issues [24].

There is growing evidence that single-stage management is superior to two-stage methods (LC with pre- or post-operative ERCP) [25]. Cholelithiasis and choledocholithiasis can be safely and effectively managed with laparoscopic transcystic CBDE. Laparoscopic transcystic CBD exploration has been successfully implemented and promoted due to technological advancements [26].

However, transductal laparoscopic bile duct exploration has been associated with increased postoperative morbidity, primarily in the form of bile leakage and, in certain cases, postoperative pancreatitis. It also leads to longer hospital stays and extended operating times [27-28]. If transcystic exploration fails, endoscopic stone extraction remains an option, either during or after the procedure.

Our study demonstrates that treating large bile duct stones associated with gallstones is safe and feasible in a single session using the transcystic approach. Combining this method with advanced technologies like the SpyGlass Discover and electrohydraulic lithotripsy (SG-EHL) can achieve effective stone removal. While a transductal approach may still be required for complete bile duct clearance, SG-EHL helps reduce the size of the bile duct opening, facilitating the removal of larger stones [18].

The SpyGlass Discover system allows for both percutaneous and laparoscopic transcystic exploration of the CBD and the removal of CBD stones. Additionally, challenging CBD stones that are unsuitable for traditional endoscopic treatment can be addressed using SpyGlass Discover-guided electrohydraulic or laser lithotripsy [15].

For example, a challenging case involved a 48-year-old patient diagnosed with 12 mm CBD stones. The planned ERCP was unsuccessful and complicated by pancreatitis and a large abdominal collection, which required percutaneous drainage. Subsequently, laparoscopic transcystic CBD exploration was successfully performed, achieving bile duct clearance [29].

Another case report highlighted using SpyGlass Discover during laparoscopic procedures for treating hepatolithiasis associated with choledocholithiasis [30].

Study limitations

Laparoscopic transcystic CBD exploration with SpyGlass Discover assistance was recently introduced to our department as a novel surgical procedure, and only a limited number of cases have been performed to date. We aim to expand our study and include more patients in the future to acquire larger sample sizes for research.

Over time, we have observed improvements in the operative time for this procedure as we have become more familiar with the ergonomics of the SpyGlass Discover catheter. Future data collection could lead to improved outcomes in terms of both operative time and cost-effectiveness.

## Conclusions

Laparoscopic CBD stone removal using the SpyGlass Discover system during cholecystectomy offers several advantages. First, laparoscopic cholangioscopy is associated with minimal risk as it does not require a papillotomy. Additionally, this procedure is easily accessible and can be fully carried out by surgeons during LC without requiring specialized endoscopic training. Another key benefit is the ability to explore the CBD in patients with altered gastrointestinal anatomy or those with difficult access to the papilla. Research has shown that a one-stage LC combined with SpyGlass Discover-assisted transcystic CBD clearance is an effective, safe, and cost-effective approach.
